# Transcriptional Regulatory Network Analysis of MYB Transcription Factor Family Genes in Rice

**DOI:** 10.3389/fpls.2015.01157

**Published:** 2015-12-24

**Authors:** Shuchi Smita, Amit Katiyar, Viswanathan Chinnusamy, Dev M. Pandey, Kailash C. Bansal

**Affiliations:** ^1^ICAR-National Bureau of Plant Genetic Resources, Indian Agricultural Research InstituteNew Delhi, India; ^2^Department of Biotechnology, Birla Institute of TechnologyMesra, Ranchi, India; ^3^Division of Plant Physiology, ICAR-Indian Agricultural Research InstituteNew Delhi, India

**Keywords:** MYB TF, co-expression, co-regulatory, abiotic stress, rice, network analysis

## Abstract

MYB transcription factor (TF) is one of the largest TF families and regulates defense responses to various stresses, hormone signaling as well as many metabolic and developmental processes in plants. Understanding these regulatory hierarchies of gene expression networks in response to developmental and environmental cues is a major challenge due to the complex interactions between the genetic elements. Correlation analyses are useful to unravel co-regulated gene pairs governing biological process as well as identification of new candidate hub genes in response to these complex processes. High throughput expression profiling data are highly useful for construction of co-expression networks. In the present study, we utilized transcriptome data for comprehensive regulatory network studies of MYB TFs by “top-down” and “guide-gene” approaches. More than 50% of *OsMYBs* were strongly correlated under 50 experimental conditions with 51 hub genes via “top-down” approach. Further, clusters were identified using Markov Clustering (MCL). To maximize the clustering performance, parameter evaluation of the MCL inflation score (I) was performed in terms of enriched GO categories by measuring F-score. Comparison of co-expressed cluster and clads analyzed from phylogenetic analysis signifies their evolutionarily conserved co-regulatory role. We utilized compendium of known interaction and biological role with Gene Ontology enrichment analysis to hypothesize function of coexpressed *OsMYBs*. In the other part, the transcriptional regulatory network analysis by “guide-gene” approach revealed 40 putative targets of 26 OsMYB TF hubs with high correlation value utilizing 815 microarray data. The putative targets with MYB-binding cis-elements enrichment in their promoter region, functional co-occurrence as well as nuclear localization supports our finding. Specially, enrichment of MYB binding regions involved in drought-inducibility implying their regulatory role in drought response in rice. Thus, the co-regulatory network analysis facilitated the identification of complex *OsMYB* regulatory networks, and candidate target regulon genes of selected guide MYB genes. The results contribute to the candidate gene screening, and experimentally testable hypotheses for potential regulatory MYB TFs, and their targets under stress conditions.

## Introduction

Plants are exposed to several environmental factors and accordingly modulate their growth and development. Excess or deficit of these environmental factors from their optimum levels adversely affect the plant growth and thus crop yield (Gao et al., 2007; Shinozaki and Yamaguchi-Shinozaki, 2007; Bansal et al., 2012). Plants respond and adapt to these cues, through various molecular, biochemical and physiological processes. These processes are regulated by transcriptional regulators which mediate the transcriptional regulation of several effector genes required for stress tolerance. Hence, understanding the regulatory hierarchy of gene expression in response to diverse environmental cues is important to improve the plant processes for enhancing agricultural production.

Systematic analysis of transcriptome data decipher regulatory networks, that helps in identification of candidate genes with certain degree of coordinated expression (Xue et al., 2012; Zhang et al., 2012; Smita et al., 2013). Correlation analyses are useful to identify co-regulated gene pairs in a signal transduction pathway as well as in identifying new candidate genes for specific processes (Gigolashvili et al., 2009; Mounet et al., 2009; Vandepoele et al., 2009). Proteins encoded by highly co-regulated genes are co-localized within the cell and often physically interact with each other. Several gene clustering methods are used to identify functionally coupled genes based on expression similarity (co-expression) levels in a given set of conditions. To study the functional association among genes “guide-gene” and “top-down” approaches are generally used in system biology study. In the guide-gene approach, genes with known functions are utilized to retrieve the correlated genes in the co-expression network, while top-down approach (non-targeted) is used to identify the local module from the large network based on network topology (Patnala et al., 2013). Further, relating these modules to functional enrichment analysis leads to the identification of gene function.

Network approach have been successfully applied in order to analyze correlated genes and hub genes using high throughput expression profiling data (Aoki et al., 2007; Yuan et al., 2008; Cramer et al., 2011; Movahedi et al., 2012). The major progress in molecular genetic analyses led to the identification of several genes and TFs that directly and/or indirectly (i.e., regulated by other pathway product) regulate the plant responses to abiotic stresses (Chinnusamy et al., 2004; Nakashima et al., 2009; Xu et al., 2011). TF genes encompass a considerable portion in plant genome, and can be grouped into different, often large, gene families on the basis of their specific DNA-binding domain. This specific DNA binding domain of TF interacts with target *cis*-elements in the promoter sequence, thereby controlling the expression of the target gene. The MYB domain containing TFs constitute one of the largest TF families in plant kingdom (Qu and Zhu, 2006). The first MYB (myeloblastosis) family of transcription factor identified was the “Oncogene” v-MYB identified in avian myeloblastosis virus (Klempnauer et al., 1982). Three v-MYB-related genes namely c-MYB, A-MYB, and B-MYB were subsequently identified in many vertebrates (Martin and Paz-Ares, 1997; Weston, 1998). *MYB* genes code for TFs with a characteristic 52 amino acid MYB motifs. These TFs contain one to four MYB domain direct repeats termed as R1, R2, R3, and R4 (Du et al., 2009). As their name implies, one R-*MYB* (*MYB*-related), two R-*MYB, three* R-*MYB*, four R-*MYB* have one, two, three, and four repeats, respectively. Each MYB domain has three regularly spaced tryptophan residues that are separated by 18 or 19 amino acid residues, and each domain form helix-turn-helix fold that is crucial for MYB TF–DNA interaction (Saikumar et al., 1990). Among these, two R-MYB (R2R3) are the richest class of *MYB* TF super-family genes in plants (Dubos et al., 2010). The MYB TFs play important role in wide range of biological processes such as cell cycle regulation (Cominelli and Tonelli, 2009), cell proliferation (Xie et al., 2010), developmental processes (Komaki and Sugimoto, 2012), hormone signal transduction (Zhao et al., 2014), and abiotic stress responses (Dai et al., 2007; Liu et al., 2011; Seo et al., 2011; Katiyar et al., 2012) in plants. Several researches have demonstrated the regulatory role especially of R2R3-*MYB* genes in various abiotic stresses responses (Pattanaik et al., 2010; Yun et al., 2010; Du et al., 2012; Zhang et al., 2012).

Advances in high throughput *omics* technologies complemented with comprehensive system biology approaches offers many ways to identify gene networks that operate in a given time or a biological processes. Several TF families have been explored for regulatory network study (Meier et al., 2008; Berri et al., 2009; Lim et al., 2010; Ouyang et al., 2012), while the MYB family network has not been explored in spite of its important roles in several biological processes. In the present study, we applied co-expression network based analysis, to dissect *MYB* transcriptional regulatory networks and their correlated links in rice. Taking into account the role of *MYBs* in diverse biological processes, we selected transcriptome data for five major processes such as developmental stages, abiotic stress response, biotic stress response, hormone signaling, and phosphorus deficiency stress response. Comprehensive correlation approach was employed to answer: (i) how *OsMYBs* network connectivity relates to the significant level of co-expression between *OsMYBs* by top-down approach; and (ii) how transcriptional regulatory network based analysis complementing with *cis*-regulatory elements relates to the putative target genes by guide-gene approach. Thus, the study revealed insight into the discovery of new links and usefulness of characterizing the interacting target genes that lead to the formation of complex transcriptional regulatory network (TRN) in plants.

## Methods

### *OsMYB* identification and their genome-wide expression profiling for top-down approach

MYB domain was retrieved by searching for PFAM-ID PF00249 (MYB domain) as a query in rice genome at TIGR (http://rice.plantbiology.msu.edu/). The non-redundant dataset of *MYB* genes identified in rice genome MSU (release 7) was used as input for further validation by domain search at the Pfam database. Only the longest splice form was selected when more than one alternative splicing sequence was found for the same locus. These analyses led to the identification of 237 non-redundant *OsMYBs* genes in our study. Further, we discarded the loci lacking MYB-DNA binding domain but annotated as MYB protein family in MSU. Finally, we identified 233 *OsMYBs* genes in rice genome and named these MYBs following the nomenclature scheme suggested for TF genes in grasses (Gray et al., 2009). Affymetrix rice arrays were downloaded from NCBI Gene Expression Omnibus (GEO) (platform: GPL2025). Total fifty Affymetrix rice arrays representing five different conditions abiotic (drought, cold, salt), biotic (Magnaporthe oryzae strain Guy11), developmental stages (embryo, endosperm, root, leaf, and seedling), phosphorus deficiency, and hormone treatment (auxin; indole-3-acetic acid, and benzyl aminopurine) with minimum of two biological replicates were retrieved. The microarray data have been retrieved from NCBI GEO under the accession number of GSE6901, GSE18361, GSE11966, GSE35984, and GSE5167 (Table S1). Original.CEL files for were normalized using RMA (Bolstad et al., 2003) a package of the statistical software R-version 2.6.1, part of Bioconductor http://www.bioconductor.org/ (R Development Core, Gentleman et al., 2004). Normalization on total signal was performed using the “Robust Multi-array Average-RMA” method. In brief, gene expression raw data analysis was done using the robust multichip analysis algorithm (RMA) and *t*-test was used to calculate the *P*-value of the expression change of each probe set in each biological perturbation. Differentially expressed genes (DEGs) were identified based on normalized signal intensities of biological replicates for each samples using the limma package (Diboun et al., 2006). Fold change of gene expression was calculated using average signal intensities of biological replicates for each sample. *OsMYBs* were considered to be significantly up/down regulated when the log of expression value is ≥1.5 with adjusted *P* < 0.05.

Mapping of probes to gene models were done by searching in the MSU Rice Genome Annotation Project release—7 (based on a new pseudomolecule assembly, Os-Nipponbare-Reference-IRGSP-1.0). Microarray data used in the study were from Affymetrix platform (GPL2025) chip containing 57,381 probe sets, each consisting of 11 pairs of 25-mers probes. The 123 probes designed for bacterial/phage control were not included in further analysis. Particularly, when we searched probes matching for *OsMYBs*—264 probe sets matched for 223 *OsMYB* loci (more than one probes matched with one loci). Out of 223 *OsMYBs*, 219 were mapped to 262 probe sets, while no probe sets for 14 *OsMYBs*. Among 219 *OsMYBs*, 183 *MYB* genes had single probe, while the remaining 36 *OsMYBs* were represented by more than one probe. To avoid ambiguity during analysis, the average expression was calculated for the genes having multiple probes.

#### Expression correlation network construction

The expression correlations assembled in matrix of all-versus-all *OsMYB* genes were calculated by Pearson correlation coefficient (PCC; *r*-value) that capture the linear relationships between any two given components. Expression correlation data were used for correlation network, where nodes represent genes and edges are correlation coefficient value among gene pair. The network was further visualized and analyzed using Cytoscape version 2.8.3 (Shannon et al., 2003).

#### Module detection, assessment and GO enrichment analysis

Highly interconnected genes were identified by best graph partitioning algorithms called Markov Clustering algorithm (MCL) (Van Dongen, 2008). The MCL algorithm is designed specifically for clustering of simple or weighted graphs. The MCL algorithm finds cluster structure in graphs by a mathematical bootstrapping procedure. Since the results of MCL depend heavily on the choice of an inflation parameter (I), we applied MCL to the networks constructed with varied I between 1.1 and 3.0 to identify the functional clusters. Clusters with less than three probesets are often biologically meaningless and were removed.

Further, the evaluation of functionally enriched were done by assessment of gene ontology (GO) term overrepresentation within a cluster, as discussed by Wong et al. (2014). Gene Ontology enrichment analysis was done by “g:Profiler” Gene Ontology enrichment analysis tool (http://biit.cs.ut.ee/gprofiler/) using the hypergeometric distribution adjusted by set count sizes (SCS) for multiple hypothesis correction (Reimand et al., 2011). SCS threshold remove enriched false positive GO terms and prioritizes truly significant results. Each probe IDs were assigned GO term, if it crossed the threshold adjusted *P*-values (SCS) < 0.05. The evaluation of cluster performance using MCL at various *I*-values was determined by calculating the fraction of modules enriched with one annotation at FDR < 0.05 (expressed as specificity) and the fraction of annotations enriched in at least one module at FDR < 0.05 (expressed as sensitivity), having at least two genes associated with the enriched annotation (Wong et al., 2013). The specificity and sensitivity values were then summarized as a functional enrichment score, the F-measure, calculated as the harmonic mean between specificity and sensitivity [(2 × Specificity × Sensitivity)/(Specificity + Sensitivity)].

#### Phylogenetic analysis

Multiple sequence alignment of full OsMYB amino acid sequences was performed by Clustal X 2.0.11 using default parameters. Rooted phylogenetic tree topologies were constructed by the Neighbor-Joining (NJ) method and the distances were obtained using a PAM-like distance matrix. The pairwise deletion and p-distance model parameters were used. Bootstrap test (1000 replicates) was performed to validate the phylogenetic tree. The phylogenetic tree image was displayed with the iTOL programme (http://itol.embl.de/; Letunic and Bork, 2011). In tree view, the branches with >1000 bootstrap were shown as green nodes, while red nodes had >80 but < 1000 bootstrap value. Most of the genes with high Bootstrap values shown the evolutionary relatedness of genes with high confidence.

### Transcriptional co-regulatory network construction and inference using guide-gene approach

The transcriptional co-regulatory network was built by RiceFREND database (http://ricefrend.dna.affrc.go.jp/) with hierarchy equal to two and mutual rank was set as five (Sato et al., 2013). The database contains 815 microarray data from various tissues at different developmental stages and plant hormone treatment conditions with the access of single and multiple guide-gene searches. In order to exclude the expression correlation due to the constitutive expression pattern, the correlated genes with weighted PCCs higher than the optimal (0.6) thresholds were only extracted from the database and considered as the putative co-expressed genes.

#### *Cis*-element enrichment analysis

PlantCARE database (http://bioinformatics.psb.ugent.be/webtools/plantcare/html/) was used to predict *cis*-regulatory elements in the promoter region (1 kb upstream from the translational start codon (Lescot et al., 2002). Over representation of *cis*-regulatory elements in promoter region (−1000 bp) were performed by de novo motif finder Multiple EM for motif elicitation tool (MEME; Bailey et al., 2006) with maximum number of motif set to five, *E* = 0.01, minimum motif width 6 and maximum motif width 10.

#### Subcellular localization prediction

Subcellular localization was predicted using consensus results of four localization predictor; Plant-PLoc (version 2) http://www.csbio.sjtu.edu.cn/bioinf/plant/ (Chou and Shen, 2008), (ii) WoLF PSORT http://wolfpsort.org/ (Horton et al., 2007), (iii) CELLO (version 2.5) http://cello.life.nctu.edu.tw/ (Yu et al., 2006), and (iv) GO slim from TIGR-MSU database.

## Results

### *OsMYB* co-regulatory network using top-down approach

#### Retrieval of *OsMYBs* and transcriptome data pre-processing

By a reiterative database exploration with Pfam-ID PF00249 as a query at TIGR, a total of 237 nucleotide sequences were retrieved from rice genome as putative *OsMYB* genes with at least one MYB domain. These candidate genes were further examined by searching for MYB domain at Pfam database. Based on this, we identified 233 MYB genes and named them following the nomenclature scheme suggested earlier (Gray et al., 2009; Table S2). Computational domain analysis of final non-redundant set of 233 *MYB* genes showed the presence of several other functional domains including WD domain, G-beta repeat, response regulator receiver domain, BTB/POZ domain, SWIRM/Zinc finger domain, and MYB-CC type transfactor (LHEQLE motif). In total, 113 *MYB*, 70 *MYB* related, 44 *G2*-like *MYB*, and 6 *ARR-B MYB* genes were identified and mapped on rice chromosomes. We observed the variant density distribution of *MYB* genes on rice chromosomes. It reflects the genome/ tandem duplication and gene amplification of *MYB* over evolutionary time.

Gene regulation in response to a physiological perturbation and those triggered by developmental stages can be inferred by appending one dataset with the other. As MYB has diverse role in stresses as well as developmental stages, we have mined and append genome wide expression data of *OsMYBs* from a total of 50 Affymetrix rice arrays for different conditions *viz.* abiotic (GSE6901), biotic (GSE18361), developmental stages (GSE11966), phosphorus deficiency (GSE35984), and hormone treatment (GSE5167; Table S1). Differentially expressed *OsMYBs* were identified based on normalized signal intensities of biological replicates for each sample. About 20% *OsMYBs* showed significant expression change (log fold ≥ 1.5; adjusted *P* = 0.05) in at least one of the experiment (Table S3). Gene Ontology enrichment analysis showed that *OsMYBs* differentially expressed were associated with genes involved in the regulation of biological process such as response to freezing, abiotic stress, endogenous stimulus, environmental stimulus, regulation of two-component signal transduction system (phosphorelay), etc., (Table S4). The transcriptional responses of *MYB* TFs to several cues clearly indicated the existence of a complex regulatory circuit comprising transcriptional activator as well as repressors. Hence, we utilized and correlated these data for understanding of regulatory network in further analysis.

#### OsMYB co-expression network construction with cross-validated expression correlations

**Figure 1:**
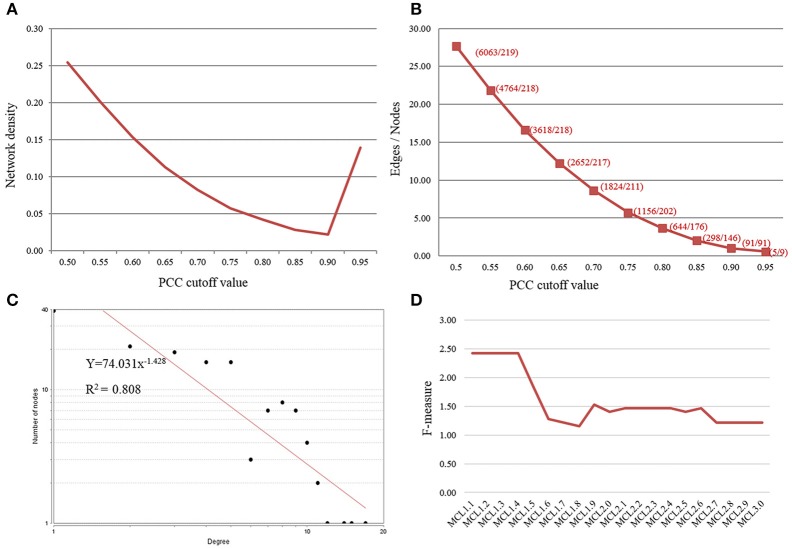
**Selection of Pearson correlation coefficient (PCC) threshold value**. **(A)** Plot of number of edges and nodes vs. PCC threshold value. **(B)** Plot of Network density as a function of PCC threshold value. **(C)** Network satisfying scale-free topology showed the node degree distribution following power law (*R*^2^ > 0.8). **(D)** Parameter evaluation and optimization of the MCL inflation score (*I*) for cluster performance by F-measure.

The complete expression data of 219 *OsMYBs* (mapped to the probsets; see Section *OsMYB* Identification and Their Genome-wide Expression Profiling for top-down Approach) was further recruited for co-regulatory network analysis. The correlations were measured using log transformed (logarithmic) expression values and co-expression network was built as well as analyzed with Cytoscape (Table S5A). The topology for networks was examined at different threshold of PCC. This showed that increasing PCC cutoff value leads to decrease in number of both nodes and edges (Figure 1A). It was observed that with increasing the PCC value from 0.85 to 0.90, the number of nodes was reduced by 37.67%, while the number of edges was dropped drastically by 69.46%. This drastic reduction in the number of edges may drop important biological interaction. Hence, to possess relatively large number of nodes and their correlation in the network, we opted 0.85 as stringent PCC cutoff value. For the topology, selecting PCC cutoff 0.85 was confirmed by plotting the number of edges, nodes, and network density as a function of the threshold values. The network density at the governed cutoff was ~0.027 in co-expression network, and increased thereafter (Figure 1B). The network created in this study satisfied the scale free topology (Figure 1C; Albert and Barabasi, 2000).

The preliminary co-expression network was constructed by connecting genes with PCC magnitude >0.85 and said to be strongly coexpressed genes (PCC > 0.85; positively co-expressed and < −0.85; negatively coexpressed) (Figure S1). Total of 146 (66.67%) *OsMYBs* and 298 correlations in network at 0.85 PCC cutoffs were obtained. Among all correlation, a total of 95.30% paired genes had positive correlation; while 4.69% paired genes had negative correlation (Table S5B). Genes with positive correlation depict the role of interacting partner in a coordinated manner in similar biological pathway, while genes showing negative correlation might be effective in opposite regulation of genes for a physiological response. This analysis revealed the existence of three major co-regulatory sub-networks with nodes having greater than 3°, in networks (Figure S1). Network analyses revealed that 151 out of 219 (68.95%) of the rice *MYB* genes analyzed in this study are coexpressed with diverse degree of connectivity with other *OsMYBs*.

#### Specificity of module with GO enrichment

**Figure 2:**
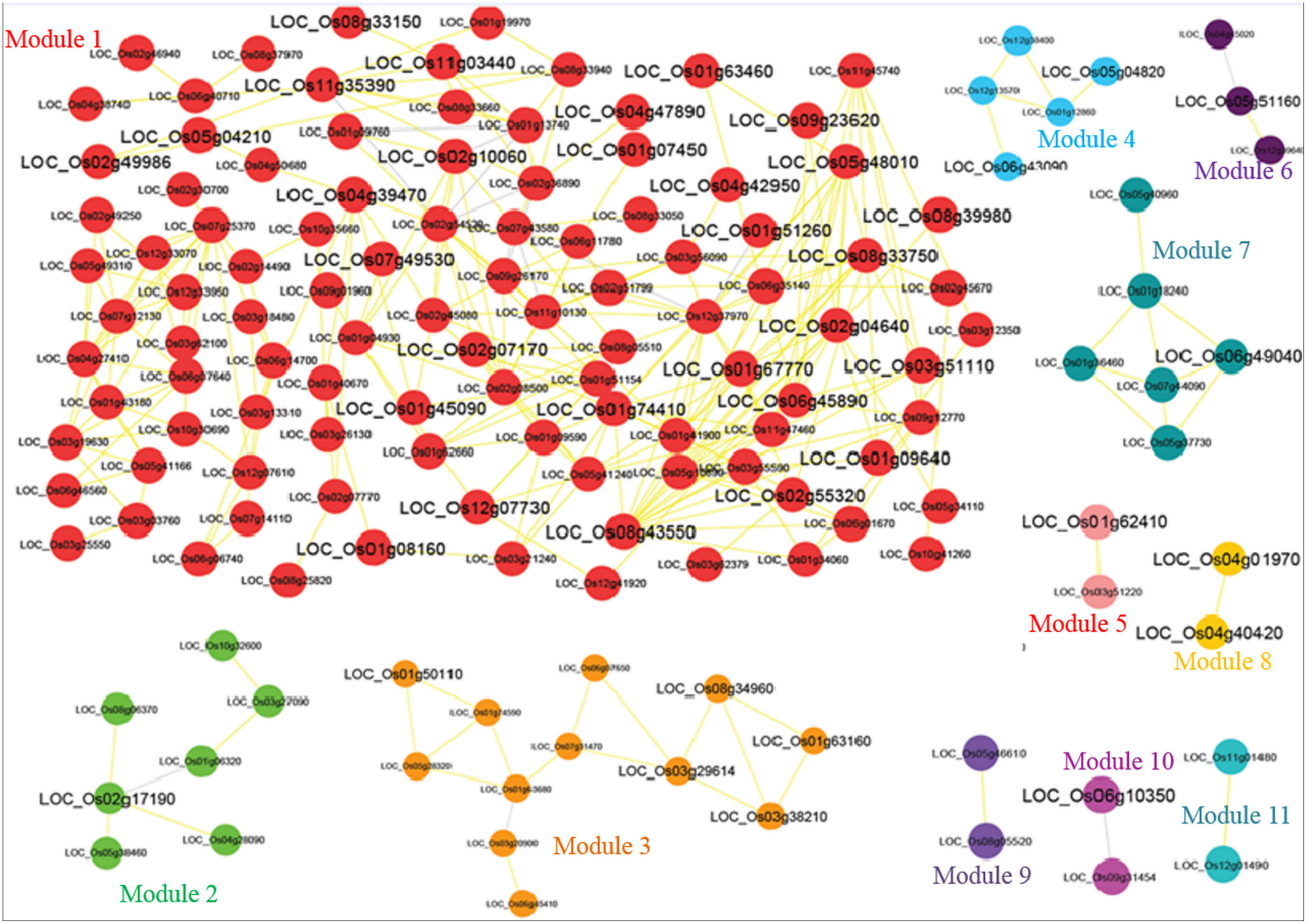
**Co-regulatory rice ***OsMYB*** network identified by top-down approach**. The 11 modules are shown in different color. Fonts in larger size indicate differentially expressed *OsMYBs*. The positive and negative correlation value is shown by yellow and gray color edges, respectively.

Grouping of the cluster of coexpressed genes into “modules” also reflects regulatory relationships found in biological systems. One can conclude the function of unknown genes through “guilt by association” with well-characterized genes. We grouped the biologically related coexpressed genes by modular analysis to unravel the underlying functional processes. Several graph clustering methods based on sharing of common functional and expression relatedness are being used in biological science. We subjected the whole *OsMYB* network for module analysis by MCL (Markov Cluster) algorithm (Van Dongen, 2008). This algorithm has an important Inflation parameter (*I*). Higher value for *I* tends to produce a large number of modules but smaller in size. Parameter evaluation and optimization of the MCL inflation score (*I*) is often necessary to maximize clustering performance (the quality of derived GO predictions based on specificity, sensitivity and F-measure; Wong et al., 2013). We examined different inflation values between 1.1 and 3.0. At inflation value 1.1–1.3, no modules were obtained. At *I* value of 1.4 onwards diverse number of modules were obtained in network. Further, relating the largest module to diverse functional categories gives clue to opt the inflation cutoff value. We observed that an MCL *I* parameter of 1.4 produced the best clustering solution in terms of enrichment significance for GO biological process (BP) of most of the cluster and highest *F*-score (see the details in Methods Section; Table S6, Figure 1D). Therefore, with the inflation value set at 1.4, MCL detected 11 modules in the network with modularity (0.256; Figure 2). As node degree distribution, the module size distribution was also observed highly skewed. The largest module had 103 nodes; whereas smallest module had two nodes with one correlated edge in the network. Distribution of hub nodes was observed to be restricted to module 1 only.

We took the modules having more than three correlated edges (i.e., six modules) for modular GO enrichment analysis. The network possesses more number of edges and confers co-regulation of genes even with large differences in expression level. We examined the significant modular GO functional enrichment analysis for six modules using g:profiler tool with cut-off using the hypergeometric distribution adjusted by set count sizes (SCS) *p* ≤ 0.05 (Figure 2). The module genes were significantly enriched in response to gibberellin stimulus (GO:0009739; g:scs < 6.94E-06), jasmonic acid stimulus (GO:0009753; g:scs < 5.54E-06), hormone stimulus (GO:0009725; g:scs < 1.17E-02), auxin stimulus (GO:0009733; g:scs < 6.27E-03), temperature homeostasis (GO:0001659; g:scs < 2.66E-04), abiotic stimulus (GO:0009628; g:scs < 5.10E-04), cold (GO:0009409; g:scs < 2.20E-03), response to freezing (GO:0050826; g:scs < 2.96E-04) etc. with highest significance. *OsMYBs* of module 3 were found to be significantly enriched with GO term positive regulation of response to stimulus (GO:0048584; g:scs < 1.04E-02). Besides, the molecular functions related to DNA binding and nucleic acid binding were significantly enriched. More detailed knowledge about the significant and unique biological processes, molecular functions, and cellular component where the *OsMYBs* act are given in Table S4.

#### Evaluating the relationship between differential expression and functional coherence of a modular *OsMYBs*

The correlation analysis gave a hint to correlate the significant relationship between regulatory modular *OsMYB* genes and the differentially expressed *OsMYBs*. To investigate this relationship between differentially expressed genes in the network, we assessed topological properties of network and function of *OsMYB* nodes and hubs (labeled in red color in Figure S1, Figure 2). We observed this kind of relationship especially in 1st, 2nd, and 7th modules. Analysis showed that more than 50% of the genes of module 1 were found to be upregulated under drought conditions. Among them, one pair of *OsMYB*; *LOC_Os09g23620* and *LOC_Os02g04640* was positively correlated (0.80) with each other. We observed that *LOC_Os02g55320* and *LOC_Os01g67770* were positively correlated (0.90) with each other and were found to be up regulated in leaf by more than two-fold with significant enrichment of two-component signal transduction system.

First module gene *LOC_Os03g51110* was found to be upregulated in leaf and down regulated in phosphorous deficiency and significantly enriched with response to organic substance. This gene positively correlated with other upregulated genes in the leaf *viz. LOC_Os08g43550, LOC_Os06g45890*, and *LOC_Os08g33750*. Most of the genes in second modules are induced in leaves, which imply that this module may serve as a tissue specific regulator in rice leaves, whereas some of them were found to be down regulated in root. *LOC_Os11g03440* showed positive correlation with *LOC_Os11g35390*. Interestingly, module 3 contained 12 *OsMYBs* that were found to be negative regulator of leaf and all these genes were found to be correlated with each other. We observed correlation of *LOC_Os01g63160* with two other *OsMYBs* viz. *LOC_Os08g34960* and *LOC_Os03g38210* genes, while *LOC_Os03g38210* correlates with *LOC_Os03g29614* and *LOC_Os08g34960*.

#### Assessment of phylogenetic conserved modules

**Figure 3:**
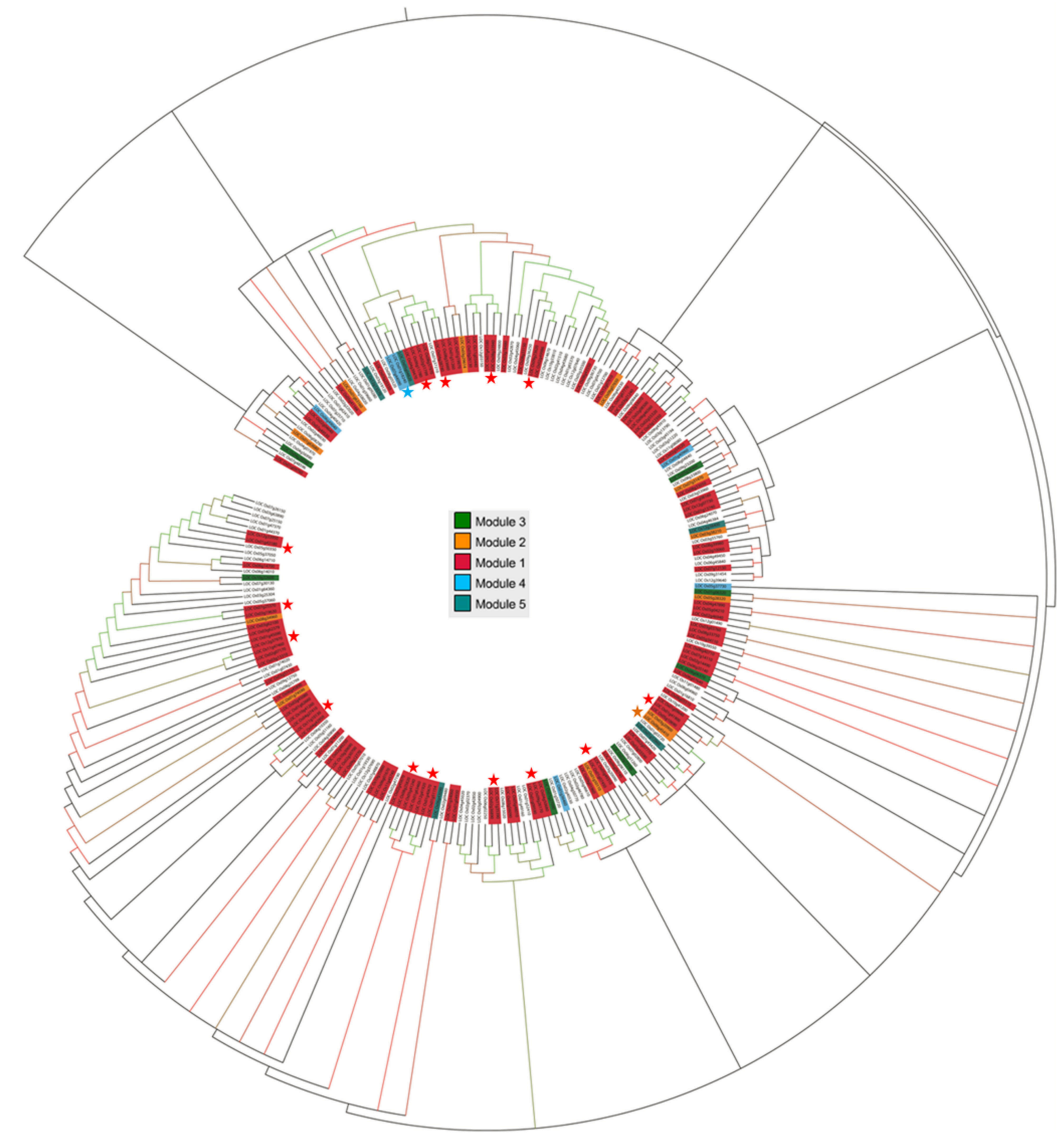
**Phylogenetic tree of OsMYB proteins**. *OsMYBs* lie in module 1 (highlighted in red), module 2 (in orange), module 3 (in green), and module 4 (in blue), were found to be evolutionary related with high bootstrap values. Gene pair marked with star (^*^) showed their sequence conservation with high boot strap value as well as coexpression which lies in same module. Bootstrap values higher than 80 are indicated by colored nodes (green nodes with >1000 bootstrap value; red nodes with >80 but < 1000 bootstrap value).

Considering the fact that the knowledge of sequence conservation is additive in identification of coexpressed gene clusters (Elnitski et al., 2006), phylogenetic analysis was performed with the Maximum Likelihood method using all OsMYB protein sequences to infer diverse conserved cluster. The tree revealed six main phylogenetic groups, which were further sub-grouped in to smaller clades based upon the bootstrap values. We then mapped the selected six functionally enriched modules (see Section Specificity of Module with GO Enrichment) on the phylogenetic tree (Figure 3). Particularly, genes lie in module 1, 2, and 3 were found to be in different clade with high bootstrap values. This illustration was signifying the sequence conservation of these modules as well as their co-regulatory roles. Majority of the network modules clearly grouped in to different phylogenetic groups suggesting that evolutionarily diverse *OsMYB*s contributing to orchestrate a specific common signal transduction pathway in a network.

All clades identified based on evolutionary relatedness showed the existence of co-expressed *MYB* genes in clusters. Moreover, some of the *OsMYBs* of module 1, 2, 3, and 4 showed strong positive correlation within the whole network module as well as sequence conservation. For example, module 1 gene *LOC_Os12g37970* had significant positive correlation (0.90) with *LOC_Os11g47460* and observed to be evolutionarily conserved in largest phylogenetic group. *LOC_Os07g44090* of module 4 had strong positive correlation (0.90) with *LOC_Os01g18240* and occupied in thirrd phylogenetic cluster. We observed that OsMYB2P-1 (LOC_Os05g04820) protein was close to LOC_Os01g65370, LOC_Os05g3550, and OsMYB4 (LOC_Os04g43680) in 3rd phylogenetic cluster. Specificity of the genes lies in one module as well as together in one phylogenetic clad suggested its evolutionary role in co-regulatory manner.

#### Hub *OsMYBs* in regulatory network exhibit biological significance

Genes with high degree of connectivity either positive/negative correlation was defined as hub genes. In this study, we defined “hubs” as nodes having five and more than five connectivity in the whole network (Patil and Nakamura, 2006; Lu et al., 2007). We found 51 *OsMYBs* as hub genes which were present in network (Table S5C). Additionally, candidate hub nodes that were significantly enriched in higher level of biological processes such as signaling were adopted as a factor for potential hub genes in the network. We observed high correlation (positive/negative) among hub nodes themselves. Among 51 hubs, 48 hub *OsMYBs* were significantly enriched with GO term, while three hub genes were not found to be enriched with any GO term. Among 48 hub *OsMYB*s, 17 were significantly enriched with response to salicylic acid stimulus, stimulus, hormone stimulus, jasmonic acid stimulus, gibberellin stimulus, and abscisic acid stimulus related GO biological processes (Table 1). Results revealed that nodes pertaining to molecular functions such as DNA binding (GO:0003677; g:scs < 4.29e-32), nucleic acid binding (GO:0003676; g:scs < 1.13e-21), two-component response regulator activity (GO:0000156; g:scs < 2.91E-02), organic cyclic compound binding (GO:0097159; g:scs < 7.12e-14), etc. The details of all 48 hub nodes and significantly enriched GO biological processes were summarized in the Table S4.

**Table 1:** **Hub ***OsMYB*** genes that were significantly enriched with abiotic stress and hormone related Gene Ontology (biological process)**.

**Hub node MSU_ID**	**Degree**
LOC_Os01g13740	8
LOC_Os01g62660	5
**LOC_Os01g67770**	10
LOC_Os02g08500	12
**LOC_Os02g10060**	7
LOC_Os02g36890	8
LOC_Os02g54520	14
**LOC_Os02g55320**	5
**LOC_Os03g51110**	6
LOC_Os03g55590	5
**LOC_Os04g39470**	5
**LOC_Os05g48010**	9
LOC_Os06g01670	5
LOC_Os06g11780	6
LOC_Os07g43580	5
**LOC_Os08g43550**	15
**LOC_Os11g35390**	5
**LOC_Os12g37970**	17

Nodes in bold are differentially expressed in at least one of the condition.

**Figure 4:**
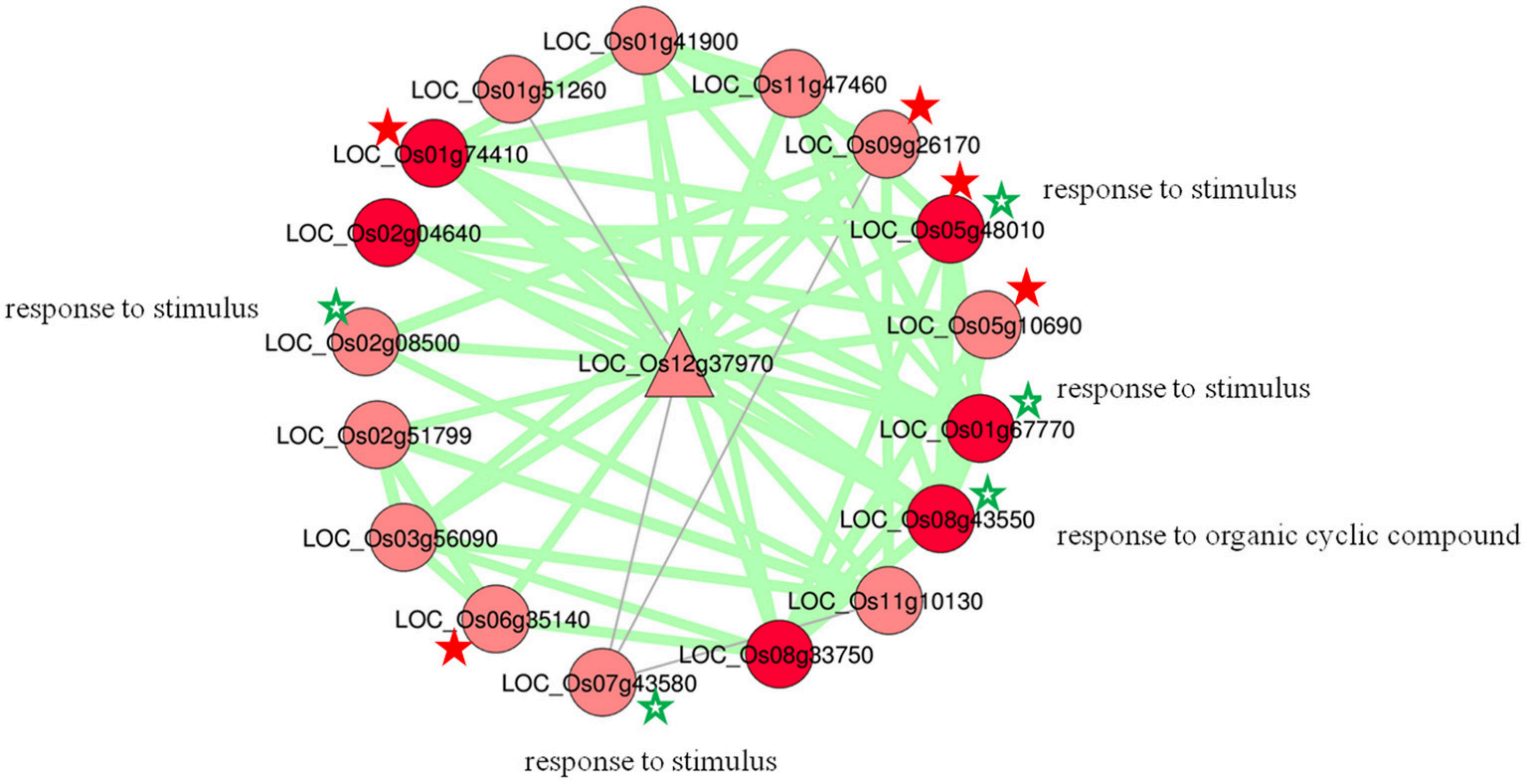
**Hub ***OsMYB***LOC_Os12g37970 with the highest connection in co-regulatory network**. Differentially expressed nodes are in dark red color. Genes with abiotic stress related GO term are marked with green star (^*^). Characterized genes having role in abiotic stress response are highlighted with red star (^*^).

The hub node *LOC_Os12g37970* with highest degree had 17 coexpressed neighbors; 15 positive and 2 negative, with an average correlation 0.88 and 0.86, respectively (Figure 4). GO analysis of sub-network of this highest degree node revealed that five nodes are significantly enriched with GO biological processes in response to stimulus and response to hormone stimulus. Among 17 coexpressed *OsMYBs*, six were found to be differentially expressed in at least one of the conditions taken in the present study. Where, three (*LOC_Os01g74410, LOC_Os11g47460*, and *LOC_Os07g43580*) were differentially expressed in our previous study under drought condition with more than 1.5-fold change (Katiyar et al., 2012). The function of individual genes was explored on the basis of GO annotation and found to be involved in endogenous stimulus, stress, abiotic, signal transduction pathways for all positively correlated genes. While two pair of genes with negative correlation; first the *LOC_Os07g43580* has role in cell death, lipid metabolic process, biotic stimulus and other one *LOC_Os01g51260* has role in flower development. These data clearly showed that the hub genes and their interacting genes as putative nodes to function in several stresses and hormones signaling pathway.

### Abiotic stress responsive *OsMYB* transcriptional regulatory network (TRN) by guide-gene approach

**Figure 5:**
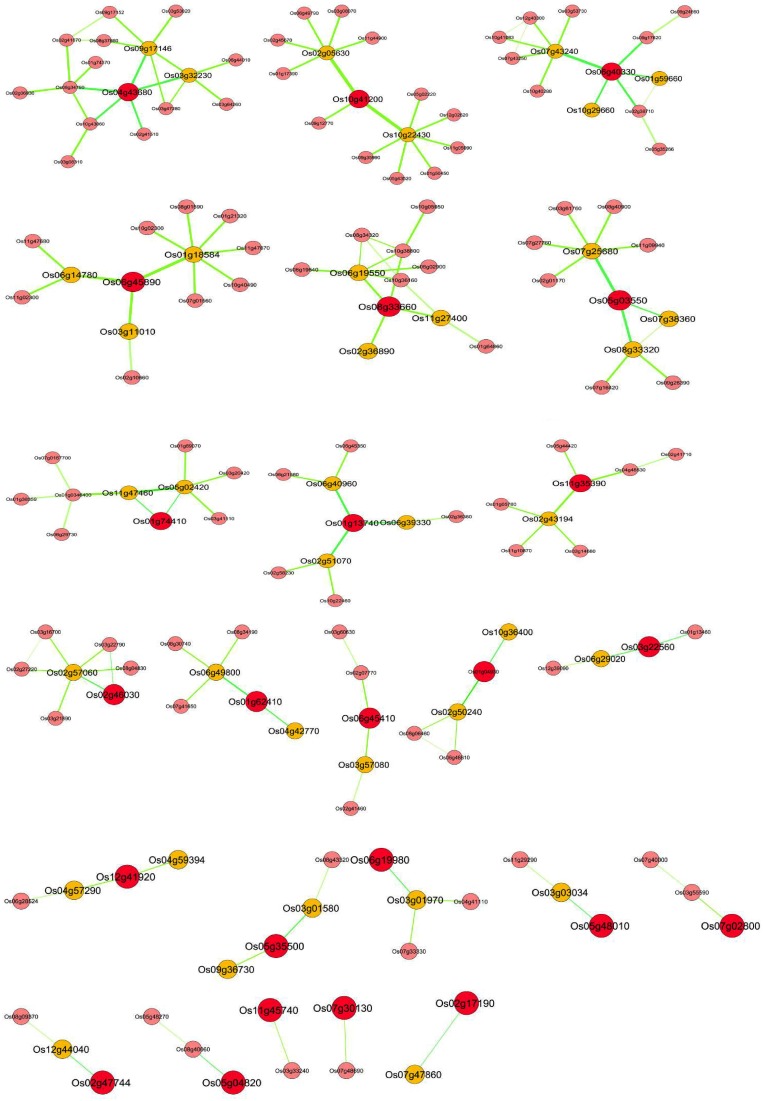
*****OsMYB*** transcriptional co-regulatory network constructed using guide-gene approach**. The co-expression network of 24 reported drought responsive genes as guide *OsMYBs* (RAP_ID, enlarged red circle); their putative first neighbor target gene (RAP_ID, orange circle) based on integrative analysis of coexpressed gene and over representation of target promoter motif enrichment with that class of transcription factor. A link between two nodes indicates direct interaction with PCC > 0.64 and MR < 10. The thickness and brightness of the edges represents the confidence of the interaction.

Identifying directly co-regulated genes (i.e., genes that are both co-expressed and share conserved upstream regulatory sequences) is important for exploring the underlying transcriptional regulatory network and putative target genes (Imam et al., 2015). For this purpose, based on the available biological knowledge, certain *OsMYBs* were selected as guide genes that are known to play key role in a specific biological process. Total of 35 *OsMYB*s were chosen as guide genes to build global co-expression network that included 17 *OsMYBs* with previously known functions and 18 *OsMYB*s with more than two fold up-regulation under drought conditions in our previous study (Katiyar et al., 2012; Table S7A). The transcriptional regulatory networks have two types of nodes namely “TFs hub” and putative target genes. We employed recently published RiceFREND co-expression tool that contains microarray data for abscisic acid, gibberellins, jasmonic acid, developmental stages, etc., for co-expressed gene identification based on mutual ranking. Since hormones play significant role in adaptive response of plants to abiotic and biotic stresses, we opted RiceFREND database with multiple guide genes search option to understand the underlying transcriptional regulatory network. The resulting regulatory networks derived from this analysis contained a total of 163 correlated nodes (TFs and putative target genes) with 158 correlations that include 24 guide genes with cutoff of weighted PCC > 0.6 and mutual rank < 5 (Figure 5; Table S7B).

The GO enrichment analysis of target genes showed that significant enrichment of biological processes such as response to abiotic stimulus (GO:0009628; g:scs < 1.25E-02), response to salicylic acid stimulus (GO:000975; g:scs < 5.36E-04), response to ethylene stimulus (GO:0009723; g:scs < 2.24E-02) response to gibberellin stimulus (GO:0009739; g:scs < 1.12E-03), etc. Interestingly as expected, the molecular function enrichment showed the term DNA binding (GO:0003677; g:scs < 1.04E-03) with highest enrichment. The cellular component showed the nucleus (GO:0005634; g:scs < 6.04E-08), intracellular organelle (GO:0043229; g:scs < 2.10E-03) with highest enrichment (Table S7C).

#### Co-regulated drought responsive putative target genes of *OsMYBs*

Most of the guide *OsMYB* genes in the network were found to be involved in drought response and hence, the coexpressed genes were analyzed for the presence of drought response (or abiotic stress related) regulatory elements in their promoters. As shown in Figure 5, transcriptional regulators based on coordinated expression and over representation of the *cis*-elements associated with the *OsMYB* in putative target genes may support our finding. For this purpose, *OsMYB* co-regulatory network was further analyzed for similar promoter *cis*-elements. A total of 53 genes as a direct neighbor of 26 guide *OsMYBs* were found. Localization prediction showed that the majority of the co-regulated MYB TF-target pairs have nuclear localization. The presence of nuclear localization signal and GO cellular location in MYB TFs and their target genes suggest that these pairs are not only co-expressed but also localized in the same cellular (nucleus) location. Further, this suggests their putative physical interactions and function in the same signaling/gene expression pathway.

The results encouraged us to identify putative targets of guide *OsMYB* genes having MYB binding *cis*-elements in their promoter region. Interestingly, we observed around 40 (75%) putative target genes with at least one MYB binding region in their promoter region (Table 2). Remarkably, among all 40 putative targets, 27 (~67%) were found to be enriched with 44 MYB binding regions involved in drought-inducibility (MBS; CAACTG, and TAACTG), implying their regulatory role in drought response. Among 27, nine were annotated as unknown proteins having MYB binding *cis*-element in their promoter. Furthermore, MYB binding site involved in light responsiveness (MRE; AACCTAA) and flavonoid biosynthetic gene regulation (MBSII; AAAAGTTAGTTA) were also found to be enriched in the putative target genes. The results suggested the multiple functionality of *MYB* targeting genes which have association with abiotic stress, function in light signaling, flavonoid biosynthesis and circadian control (Kuno et al., 2003; Dubos et al., 2010).

**Table 2:** **The guide ***OsMYB*** genes and their first neighbor as putative target with MYB binding ***cis***-elements within 1 kb upstream promoter region**.

**Guide gene**	**First neighbor gene; PCC**	MYB binding related *Cis*-elements^*^	**Strand**	**Position**
Os04g43680 (MYB family transcription factor, OsMYB4)	Os03g32230 (ZOS3-12—C2H2 zinc finger protein); 0.7	TAACTG	+	521
		CAACTG	+	566
	Os09g17146 (unknown protein); 0.7	TAACTG	+	582
		CGGTCA	−	941
		TAACTG	−	688
		AACCTAA	−	497
Os10g41200 (Transcription factor MYBS3, OsMYBS3)	Os02g05630 (protein phosphatase 2C, putative); 0.7	TAACTG	+	729
	Os10g22430 (gibberellin response modulator protein); 0.7	CAACTG	−	641
Os06g45890 (MYB family transcription factor)	Os01g18584 (WRKY9); 0.8	CAACTG	+	27
	Os03g11010 (natural resistance-associated macrophage protein); 0.7	TAACTG	+	51
		CAACTG	+	516
		TAACTG	+	128
	Os06g14780 (unknown protein); 0.7	TAACTG	−	754
Os06g40330 (GAMYB-like1)	Os01g59660 (GAMyb); 0.7	CGGTCA	+	222
		CGGTCA	−	479
	Os10g29660 (TFIID, TATA-binding protein); 0.7	CAACTG	−	115
	Os07g43240 (SKP1-like protein 1B); 0.7	TAACTG	−	191
		CAACTG	−	286
Os05g03550 (MYB family transcription factor)	Os07g25680 (protein kinase domain containing protein); 0.7	CAACTG	+	905
		AACCTAA	+	757
	Os07g38360(unknown protein); 0.7	CAACGG	+	691
	Os08g33320 (unknown protein); 0.7	AACCTAA	−	210
Os08g33660 (MYB family transcription factor)	Os02g36890 (MYB family transcription factor); 0.6	CGGTCA	−	377
	OS10g38800 (leucine-rich repeat transmembrane protein kinase); 0.7	CGGTCA	−	196
		CGGTCA	+	360
	Os11g27400 (Glycoside hydrolase); 0.7	CAACTG	+	16
		TAACTG	−	358
		TAACTG	−	278
	Os06g19550 (Short-chain dehydrogenase/reductase SDR domain containing protein); 0.7	CGGTCA	−	377
Os01g74410 (MYB59)	Os11g47460 (MYB family transcription factor); 0.8	CAACGG	−	364
		TAACTG	−	61
		CAACTG	+	790
	Os05g02420 (unknown protein); 0.8	CAACGG	−	314
Os01g13740 (MYB family transcription factor)	Os06g39330 (UDP-glucuronosyl/UDP-glucosyltransferase family protein); 0.7	AACCTAA	+	257
	Os06g40960 (ZOS6-05 - C2H2 zinc finger protein); 0.7	TAACTG	+	77
	Os02g51070 (Starch synthase isoform zSTSII-2); 0.7	CAACGG	+	525
		CAACTG	−	288
Os11g35390 (MYB family transcription factor)	Os02g43194 (Aldehyde dehydrogenase); 0.7	CGGTCA	+	124
		CAACTG	+	555
		CGGTCA	−	267
		TAACTG	−	909
Os02g46030 (OsMyb1R)	Os02g57060 (OsCttP2 - Putative C-terminal processing peptidase homolog); 0.8	CAACGG	+	792
Os01g62410 (OsMYB3R−2)	Os04g42770 (unknown protein); 0.6	CAACGG	−	326
		CAACGG	+	345
		TAACTG	+	413
		CAACTG	−	491
		CGGTCA	+	458
		TAACTG	−	823
	Os06g49800 (ubiquitin interaction motif family protein); 0.6	CAACTG	−	744
		CAACTG	+	755
Os06g45410 (MYB family transcription factor)	Os03g57080 (PLA IIIA/PLP7, Patatin-like phospholipase family protein); 0.6	CGGTCA	−	141
		CAACTG	+	921
Os01g04930 (OsMYB2)	Os10g36400 (GIL1); 0.6	TAACTG	+	808
	Os02g50240 (glutamine synthetase, catalytic domain containing protein); 0.7	TAACTG	−	471
Os03g22560 (MYB family transcription factor)	Os06g29020 (retrotransposon protein); 0.6	CAACGG	+	515
		CGGTCA	+	189
Os06g19980 (MYB family transcription factor)	Os03g01970 (THO complex subunit 1); 0.8	TAACTG	−	555
		CAACTG	+	689
Os05g35500 (MYB family transcription factor)	Os09g36730 (P-type R2R3 Myb protein); 0.6	CAACTG	−	75
	Os03g01580 (unknown protein); 0.6	CAACTG	−	75
Os12g41920 (Similar to Single myb histone 6)	Os04g59394 (unknown protein); 0.7	TAACTG	+	67
	Os04g57290 (OsFBX153 - F-box domain containing protein); 0.6	TAACTG	−	700
		CAACTG	+	925
Os02g47744 (MYB family transcription factor)	Os12g44040 (transposon protein); 0.7	TAACTG	−	797
		AAAAGTTAGTTA	+	786
Os05g48010 (OsMYB55)	Os03g03034 (flavonol synthase/flavanone 3-hydroxylase); 0.6	TAACTG	+	533
Os07g30130 (Myb, DNA-binding domain containing protein)	OS07g48690 (DUF630/DUF632 domains containing protein); 0.7	TAACTG	−	328
Os02g17190 (Myb, DNA-binding domain containing protein)	Os07g47860 (tRNA synthetase); 0.7	CAACTG	+	286

* Seven types of MYB binding cis-elements were present—CAACGG, (CCAAT-box; MYBHv1 binding site); AACCTAA, (MRE; MYB binding site involved in light responsiveness); MBSII, (AAAAGTTAGTTA; MYB binding site involved in flavonoid biosynthetic genes regulation); TAACTG, (MBS; MYB binding site involved in drought-inducibility); CAACTG, (MBS; MYB binding site involved in drought-inducibility); CGGTCA, (MBS; MYB Binding Site).

**Figure 6:**
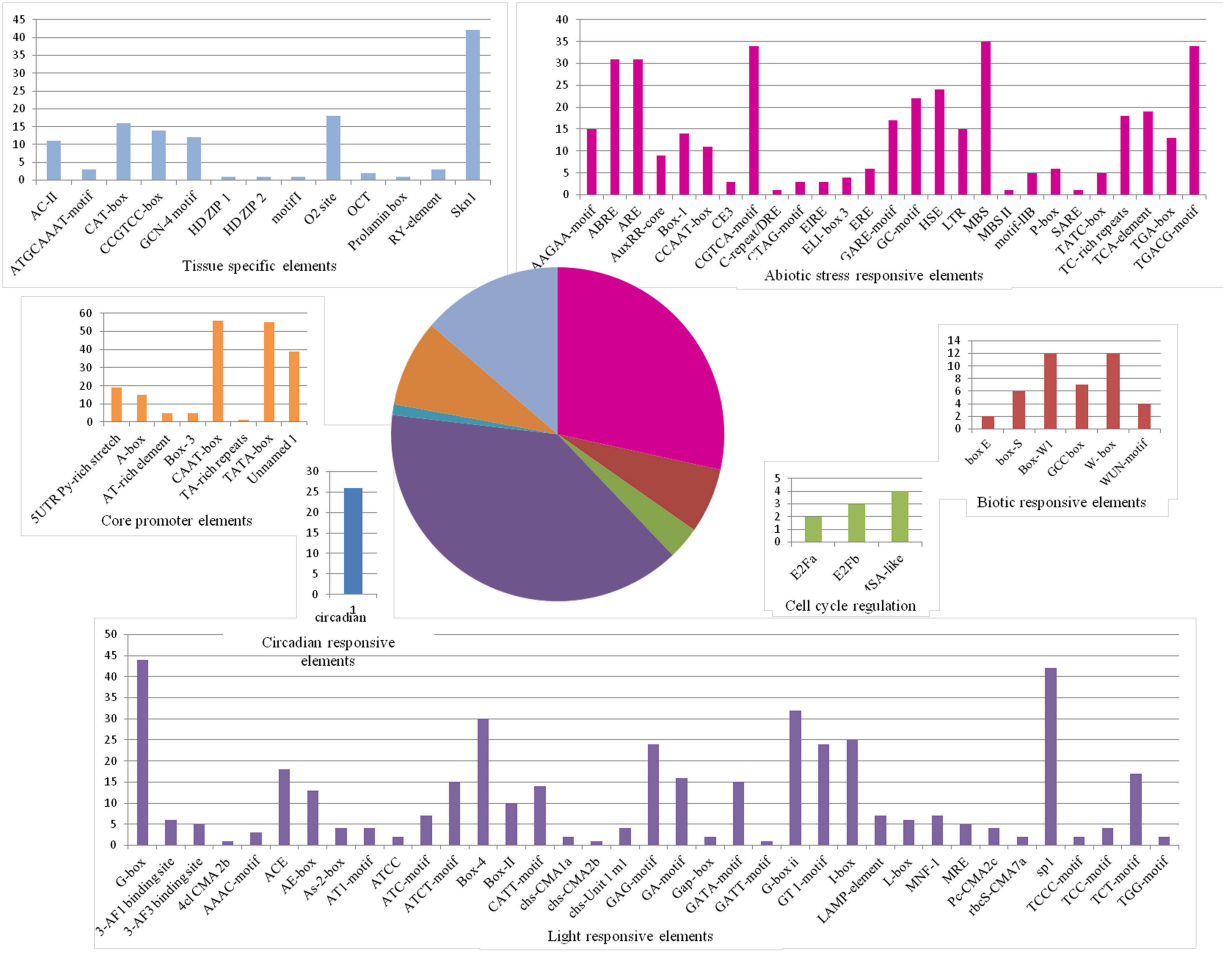
**Frequency of ***cis***-regulatory elements in the 1 kb promoter of first neighboring target genes of guide ***OsMYBs*** in the co-regulatory network**. Pie chart depicts the categorized seven types of *cis*-regulatory elements and the corresponding colored bar chart depicts the occurrence of different *cis*-elements.

**Figure 7:**
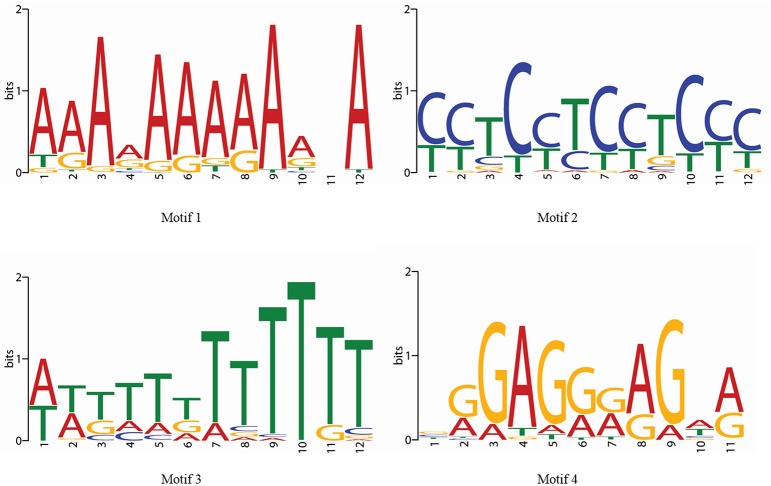
**Four enriched motifs logo in the 1 kb promoter region of first neighboring target genes of guide ***OsMYBs*** in the co-regulatory network**.

Along the MYB binding site involved in these processes, several other *cis*-elements were also found in good frequency. We categorized all the *cis*-elements in the seven broad categories on the basis of responsiveness for any perturbation (Figure 6). We observed the enrichment of light, abiotic stress and tissue specific *cis*-elements in the promoter region of first neighbor target of guide *OsMYBs*. Detailed promoter content has been summarized in Table S8A. Furthermore, the position of 44 MYB binding region involved in drought-inducibility revealed distinct patterns of sites related to proximal/distal location with respect to transcription start site (TSS). Majority of them (up to 75%) are far from TSS (~200 bp) indicating their distal type of gene expression regulation. Furthermore, the enrichment analysis of motif in 1000 bp promoter region performed by using MEME with minimum motif width 8 and maximum motif width 10 with *E*-value set to 0.01 (Table S8B). Results showed that four motifs were highly conserved in 186 sites in maximum of the target promoter sequences (Figure 7). Interestingly, we found CIRCADIAN CLOCK ASSOCIATED 1 (CCA1) motif which has been reported to be binding region of CCA1 MYB-related transcription factor (Wang et al., 1997). It supports our findings that these target genes identified in global co-regulatory network are putative and a researchable area in future.

Consideration of the phylogenetic conservation of binding sites of the promoter elements can enhance the accuracy and have a higher likelihood of being functional *in vivo* (Elnitski et al., 2006). This approach relies on the principle that biologically important TF-binding sites are more likely to be conserved during evolution (Harbison et al., 2004; Dieterich et al., 2005). Therefore, relationship between phylogenetically conserved 1 kb promoter region of all correlated gene pair in the global network and modules were investigated (Figure S2). Results showed the evolutionary conservation of several pair of correlated genes. Co-regulated genes with MYB binding regions were examined for evolutionary conservation. Results showed the presence of putative target genes having MYB binding *cis*-element from module 2; 6–10 were evolutionarily conserved. Thus, the analysis performed via top down and guide gene approaches in this study identified the highly correlated hub *OsMYBs* and drought responsive putative target genes of *OsMYBs*. Several uncharacterized hub genes as well as co-expressed genes with guide genes annotated as unknown proteins in co-expression network represent high confidence candidate regulator awaiting further examination and validation *in vitro*.

## Discussion

### Inferring function of candidate *OsMYBs* in co-expressed modules

In this study, we carried out transcriptome analysis of *OsMYB* gene family in different abiotic, biotic, hormone stress and developmental stages to identify underlying regulatory network. The *OsMYBs* were first analyzed for their differential expression and putative functions. We found, *OsMYBs* differentially expressed were associated with genes involved in the regulation of biological process such as response to freezing, abiotic stress, endogenous stimulus, environmental stimulus, regulation of two-component signal transduction system (phosphorelay), The two-component system has been shown to play an important role in response to environmental stimuli and growth regulation (Hwang and Sheen, 2001; Du et al., 2007).

The subset of genes that are differentially expressed in particular sample are also observed to be correlated with each other in a co-expression network (Cho et al., 2012). In the *OsMYBs* network of co-expressed genes identified, from the function of known gene in the network, the potential function the co-expressed genes may be inferred and could be selected as candidates for functional verification by *in vivo* approaches. The preliminary gene network of *OsMYBs* was constructed with the relative stringent thresholds to reduce false connections. Module identification and comparison with DEGs showed, correlated *OsMYB* pair in 1st, 2nd, and 7th modules was also differentially regulated under any stress conditions taken in consideration (Figure 2). GO enrichment assessment of the modules revealed the significant enrichment of term related to abiotic stress related responses. Some of the candidate genes correlating with already characterized genes for a particular condition showed their role in similar biological pathways as extracted by GO analysis also. Taken together, the coexpression results largely confirm results from previous studies and provided additional clues into the complex molecular mechanism of *OsMYBs*. *OsMYB3R-2* (*LOC_Os01g62410*) was found to be differentially expressed in drought and had positive correlation with *LOC_Os03g51220* which was found to be involved in biosynthetic process. *OsMYB3R-2* is known to confer tolerance to freezing, drought, and salt stresses in transgenic Arabidopsis (Ma et al., 2009). Several predicted *OsMYBs* were activated at early response mechanism in chilling stress (Yun et al., 2010).

*LOC_Os06g45410* positively correlated with *LOC_Os03g20900* and has role in biosynthetic processes (Table S5B). In a previous study, it was shown that *LOC_Os03g20900* has a positive correlation (0.80) with *OsATG6a* which is involved in abiotic stress (heat, cold, and drought) and abscisic acid responses (Rana et al., 2012). The *MYB* genes have been studied for their cross talk in abiotic stress and hormone regulated gene expression (Peleg and Blumwald, 2011). ABA and auxin responses were regulated by ABI5-like1 (ABL1), a bZIP transcription factor, and the expression of *LOC_Os05g04820* was changed in *abl1* mutant (Yang et al., 2011). In our study, we observed its positive correlation with *LOC_Os01g12860*. A large number of TFs interact with calmodulin (CaMs) to mediate both biotic and abiotic stress responses (Laluk et al., 2012). Recently, several putative *OsMYBs* have been reported to interact with calmodulin (Chantarachot et al., 2012). In our study, we found correlation of CaM binding MYBs i.e., *LOC_Os05g04210, LOC_Os11g45740* and *LOC_Os01g45090* with other *OsMYBs*. GO slim analysis revealed that the participation of first two genes (*LOC_Os05g04210* and *LOC_Os11g45740*) in response to abiotic stimulus and all trios in response to endogenous stimulus. In consistent with previous study, several *OsMYBs* of module were previously shown to play significant role in activation of immune response, regulation of response to stress as well as in defense response signaling pathway (Glazebrook, 2001). Module 1 genes pair were upregulated in leaf and significant enrichment of two-component signal transduction system. The two-component signal transduction system plays central role in cytokinin signaling and growth (Skerker et al., 2008; Schaller et al., 2011). Recently, it has been reported that the substantial difference in hormone signaling in several response regulators due to variation within their MYB-like DNA binding motif (Tsai et al., 2012). Hence, the correlated *OsMYB* genes may be good candidates for functional characterization of their role in abiotic stress and hormone responses.

Further identifying the hub nodes showed 51 hubs *OsMYB* in our study. These hub genes might have important roles in organizing the functional modules (Barabási and Oltvai, 2004). Some of the high degree functionally characterized hub genes such as *OsMYBS1* (*LOC_Os01g34060*), *OsMYBS2* (*LOC_Os10g41260*), and *OsMYBS3* (*LOC_Os10g41200*) have been studied previously and found to mediate sugar and hormone regulation of α-amylase gene expression (Lu et al., 2002). Moreover, *OsMYB3* is known to be essential for conferring cold tolerance to rice plants (Su et al., 2010). Another *OsMYB55* (*LOC_Os05g48010*) with 9° has been shown to confer high temperature stress tolerance and modulation of amino acid metabolism (Wahid et al., 2007). A highest hub node *LOC_Os12g37970* with 15 positively coexpressed MYB genes with their enriched GO terms “response to stimulus” and “hormone stimulus” as well as differential expression pattern suggest their function in stress and hormone signaling pathway (Figure 4). Where two negatively coexpressed OsMYBs with the hub genes showed their function in flower development, cell death and lipid metabolic process. That shows, environmental stress lead to the modulation in flower development and cell death might be due to (reactive oxygen species) ROS formation (Petrov et al., 2015).

Interestingly, we look at numerous scientific reports demonstrated the characterized genes in stress signal pathways from this highest hub cluster (Figure 4). Some of the correlated OsMYBs with this highest hub genes such as *LOC_Os01g74410* has been characterized for significant improvement in tolerance to drought and salinity stresses in rice (Xiong et al., 2014). The ortholog of *LOC_Os01g74410* i.e., TaMYB13-1 was also evidenced as transcriptional activator for fructan synthesis that known as protecting agent for drought and cold stress (Xue et al., 2011). The other coexpresssed *LOC_Os01g51260* corresponds to the *Arabidopsis* MYB TF *AT3G13890* that known to be activator of secondary wall thickening (Yang et al., 2007) and *LOC_Os08g33750* ortholog in maize for ethylene-induced lysigenous aerenchyma formation under aerobic conditions (Takahashi et al., 2015). Another positive correlated *OsMYB LOC_Os09g26170* was recently study for their significant role in MG-response and stress-responsive signal transduction pathways. (Kaur et al., 2015). Remarkably, two of the correlated 561 genes *LOC_Os05g10690* and *LOC_Os05g48010* were patented for enhancing yield-related traits in plants by modulating expression in a plant (Molinero, 2013). Hence, we hypothesize this high hub gene cluster have specific role in regulation of stress tolerance, in particular in defense mechanism as well as in crop yield improvement. And thus characterization of some uncharacterized MYB TF from this cluster can be a promising future direction.

### Phylogenetically preserved *OsMYBs* reveals strong associations between genes co-expression, function and evolution

The phylogenetic footprinting might be additive to coexpressed cluster and successfully being applied to determine expression association of genes (Elnitski et al., 2006). Exploring the co-expression and phylogenetic analysis suggested that the highly co-expressed genes with known role in specific regulatory processes were preserved in the network. We found such type of relation in module 1, 2, 3, and 4 (Figure 3). Two of the *OsMYB2* (*LOC_Os01g18240, LOC_Os05g04820*) genes were found to be upregulated in phase-I of chilling stress, where *OsMYB2* (*LOC_Os01g18240*) positively correlated with *LOC_Os07g44090* (phylogenetically also closely related), *LOC_Os05g40960, LOC_Os01g36460* and *LOC_Os06g49040*. The phylogenetically close pair was found to be involved in highly similar type of processes such as response to biosynthetic process, endogenous stimulus, reproduction, post-embryonic development. Two of the genes with high degree *viz. LOC_Os01g74410 (MYB59)* and *LOC_Os01g51154 (R1-MYB)* were found to be highly correlated with several other *MYB* genes in the network (Table S5B). It is in agreement with the study that the expression of these genes are modulated both by cold independent conditions (Park et al., 2010). We observed that OsMYB2P-1 (LOC_Os05g04820) protein was close to LOC_Os01g65370, LOC_Os05g3550, and OsMYB4 (LOC_Os04g43680) in 3rd phylogenetic cluster. OsMYB2P-1 is known to regulate phosphate starvation, cold, salt and osmotic stress responses, and also found to be up-regulated in phosphorus starvation in this study. This is in agreement with the results by Dai et al. (2012). A system biology approach has identified R2R3 motif *MYB28* and two homologs, *MYB29* and *MYB76* genes that form a single clade with distinct and overlapping functions in regulation of aliphatic glucosinolates (Sønderby et al., 2007). These evidences showed the important regulatory roles of MYBs in several biological processes. Moreover, *OsMYB4* is known to express in cold-mediated and cold-independent transcriptional network (Park et al., 2010). Evaluation of data revealed that the cluster of genes that are co-expressed lie in distinct phylogenetic clade, suggesting functional redundancy and their evolution by recent duplication.

### Deciphering transcriptional regulatory network for putative target gene identification

The first step in gene regulation is transcriptional regulation which is governed by the recognition of *cis*-element by the DNA binding domain of TFs. The assembly of TFs on the promoter *cis*-element region and their interaction in regulatory network profoundly influence the target gene expression. It is known that genes with similar expression pattern in the same biological function are likely to be regulated by same TF(s) (i.e., co-regulated) having similar *cis*-regulatory elements for the TFs were liable for putative target gene identification (Wang and Stormo, 2003; Walhout, 2006; Wang et al., 2009; Imam et al., 2015). Hence, we created another *OsMYB* network by guide gene approach to identify the putative target *OsMYB* genes on the basis of functional co-occurrence as well as MYB recognition *cis*-elements in their promoter region.

Among TFs, we observed ten guide *OsMYBs* were in correlation with other *OsMYB* genes forming a more complex feedback network. We also observe the presence of feedback motif in the target OsMYBs Comparing the results from both top-down and guide-gene approach showed the conservation of one correlated pair of *OsMYB* (*LOC_Os11g47460, LOC_Os01g74410*; PCC 0.98). Among correlated TFs such as WRKY, ZOS6-05—C2H2 zinc finger protein and helix–loop–helix (bHLH) protein were found. This suggests that the function of OsMYB proteins might require participation of various members of these transcription factors (Table S7B). It is in partial agreement with the recent study that showed transcriptional regulation by MYB–bHLH–WD40 (MBW) complex in the late step of flavonoid biosynthetic pathway (Hichri et al., 2011), GL2 expression and the non-hair or trichome fate (Schiefelbein, 2003).

Conclusively, in the present study we identified co-regulatory network and functional co-occurrence of modules of *OsMYB* genes in rice. This will contribute to illustrate the functions of gene cooperation pathways that have not yet been identified by classical genetic analyses. In the first part of the study, we adopted the top-down approach to decipher the *OsMYBs* with correlated expression pattern in different development and stress conditions. We defined the existence of *OsMYBs* gene clusters comprising both phylogenetically related and unrelated genes that were strongly coexpressed, signifying their evolutionary role in co-regulatory manner. A sum of 51 most highly connected hub *OsMYB*s were identified, some of them were expected to play the significant regulatory roles in abiotic stress tolerance. As the hubs have high correlation value, they may play crucial role in stress tolerance as well as development.

More importantly, our analyses revealed the existence of *OsMYBs* transcriptionally co-regulatory networks by taking guide *OsMYB* genes with known function under abiotic stress condition. This provided insight into the functional association of several uncharacterized genes and coexpressed putative target genes possessing MYB binding *cis*-elements in their promoter region. The presence of drought responsive MYB binding *cis*-elements in the putative target genes and guide genes with known drought stress response identified the co-regulatory network in response to drought stress. In several instances, these rationales for candidate gene screening and functional validation allowed us to generate hypotheses, which are experimentally testable and their relevance in a specific process involved in plant response to stress or hormone signals. Functional testing of *in vivo* interaction or action of the candidate co-expressed gene network modules and hubs will significantly enhance our knowledge on the function of MYB family and help develop improved rice genotypes. Therefore, the network modules predicted in the present study were of high biological relevance and revealed putative role for uncharacterized genes. Further, the outcome of the study offers new biological insights into the transcriptional regulatory networks that await experimental validation.

## Author contributions

SS, VC, KB conceived and designed the experiments. SS performed the experiments. SS analyzed the data. AK performed computational analysis. SS, VC, DP, KB wrote the paper. All authors read and approved the final manuscript.

### Conflict of interest statement

The authors declare that the research was conducted in the absence of any commercial or financial relationships that could be construed as a potential conflict of interest.

## Supplementary material

Figure S1 ***OsMYB* correlation network viewed in Cytoscape**. Nodes in red color are differentially expressed at least in one condition.

Figure S2 **Phylogenetic tree of 1 kb promoter sequences of guide *OsMYB* genes and their putative target genes**. Putative target genes having MYB binding *cis*- elements in their promoter region are shown by italic fonts.

Table S1 **Detailed list of affymetrix rice genome microarray datasets used for *OsMYB* genes correlation network analysis via top-down approach**.

Table S2 **List of retrieved *OsMYB* genes with their putative function**.

Table S3 **Differentially expressed *OsMYB* genes under diverse microarray experiments**.

Table S4 **Modular gene ontology enrichment analysis**.

Table S5 **Average logarithmic (A) signal values of 219 *OsMYB* protein encoding genes expressed under different microarray experiments**. (**B**) Pearson correlation coefficient (PCC) among *OsMYB* genes in top down approach. (**C**) Simple and complex topological properties of correlation network of *OsMYB* genes. Red highlighted is hub nodes.

Table S6 **Parameter evaluation and optimization of the MCL inflation score (*I*)**.

Table S7 **(A) List of guide genes used to create global co-regulatory network via guide-gene approach**. (**B**) Global co-regulatory network of guide *OsMYB* genes and their correlated allies with their description. (**C**) Gene ontology enrichment analysis of target genes.

Table S8 **(A)List of *cis*-elements in 1 kb upstream promoter region of direct first neighbor of guide *OsMYB* genes in global co-regulatory network. (B)** Motif enrichment analyses by MEME of direct first neighbor of guide *OsMYB* genes in global co-regulatory network.
