# Supplementary figures and images for: Transcriptional Regulatory Network Analysis of MYB Transcription Factor Family Genes in Rice
Source: Front Plant Sci. 2015 Dec 24;6:1157. doi: 10.3389/fpls.2015.01157 (PMC4689866; doi:10.3389/fpls.2015.01157)

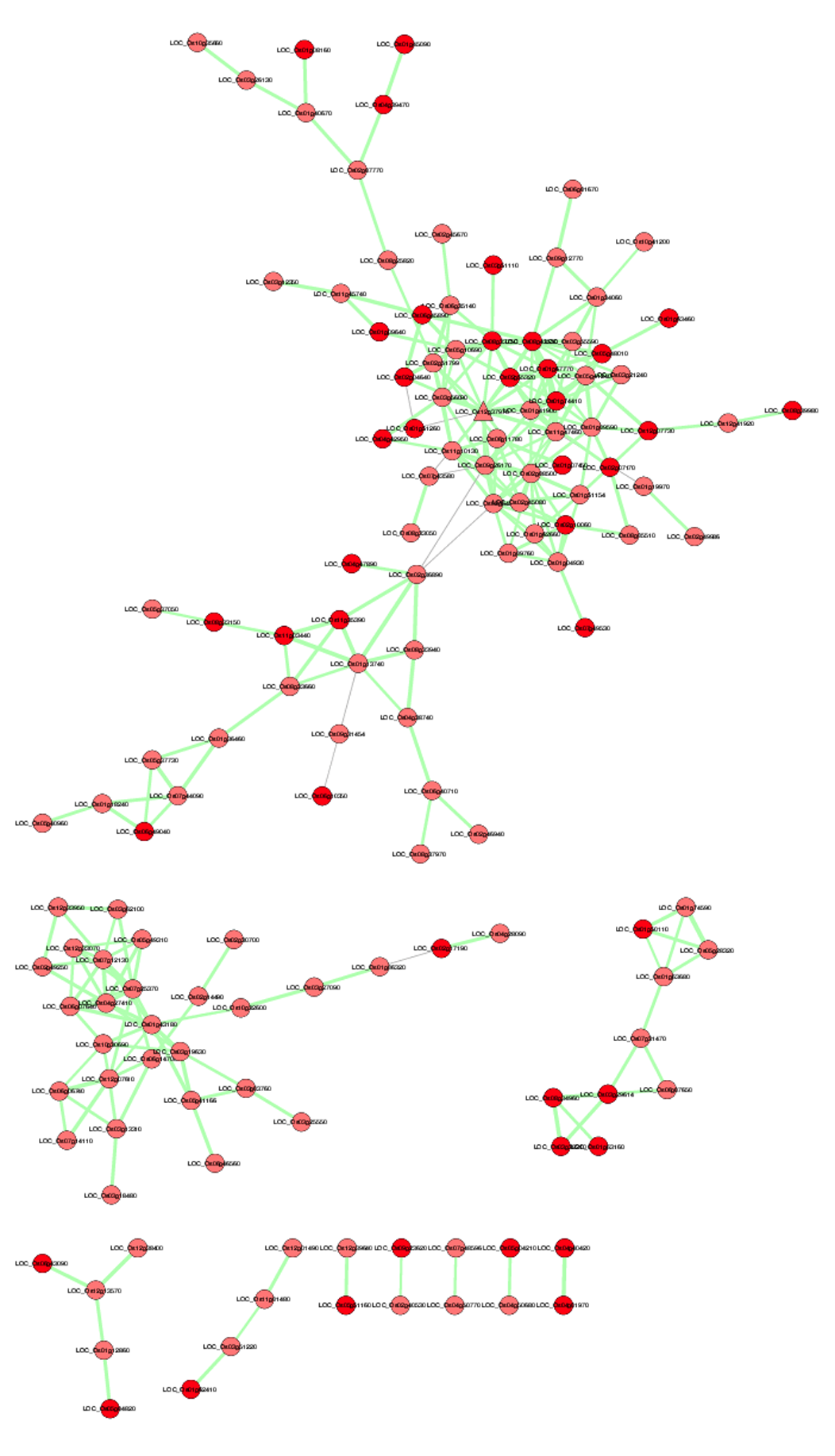

Supplement: Figure S1 — OsMYB correlation network viewed in Cytoscape. Nodes in red color are differentially expressed at least in one condition. [file Image1.TIFF]

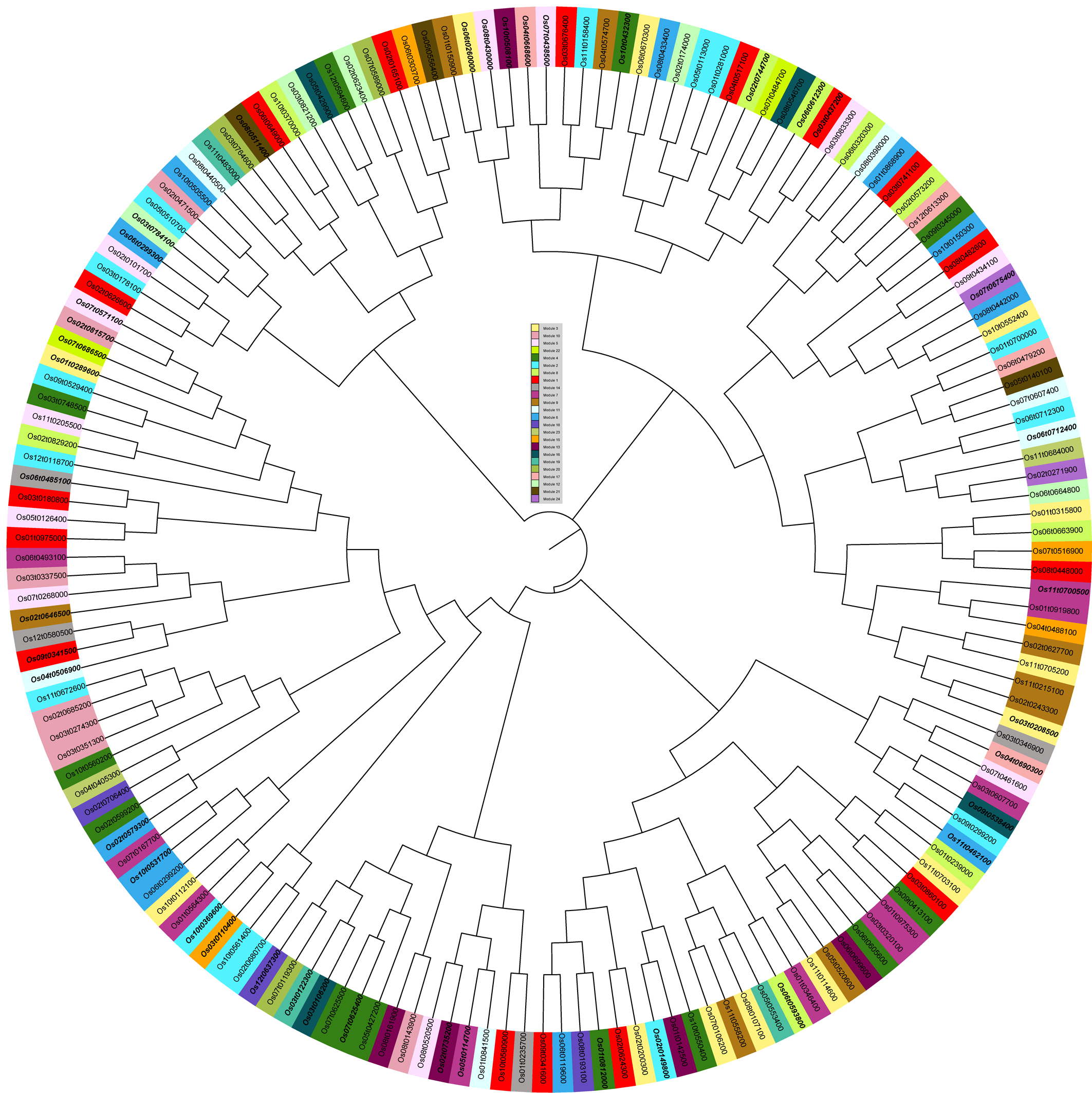

Supplement: Figure S2 — Phylogenetic tree of 1 kb promoter sequences of guide OsMYB genes and their putative target genes. Putative target genes having MYB binding cis- elements in their promoter region are shown by italic fonts. [file Image2.TIF]
